# Characterizing ligand-receptor interactions and unveiling the pro-tumorigenic role of CCL16-CCR1 axis in the microenvironment of hepatocellular carcinoma

**DOI:** 10.3389/fimmu.2023.1299953

**Published:** 2024-01-11

**Authors:** Zongbo Dai, Yu Wang, Ning Sun, Chengshuo Zhang

**Affiliations:** ^1^ Hepabobiliary Surgery Department, First Hospital of China Medical University, Shenyang, China; ^2^ Department of General Surgery, Anshan Central Hospital, Anshan, China

**Keywords:** ligand-receptor interaction, macrophages, CCL16, CCR1, hepatocellular carcinoma

## Abstract

**Background:**

The heterogeneity of the tumor microenvironment significantly influences the prognosis of hepatocellular carcinoma (HCC) patients, with cell communication through ligand-receptor complexes playing a central role.

**Methods:**

We conducted single-cell transcriptomic analysis on ten HCC tissues to identify ligand-receptor genes involved in malignant HCC cell communication using CellChat. Leveraging RNA-Seq data from the TCGA Liver Cancer (TCGA-LIHC) and Liver Cancer - RIKEN, JP (LIRI-JP) cohorts, we employed Cox regression analysis to screen for prognosis-related genes. Prognostic risk models were constructed through unsupervised clustering and differential gene expression analysis. Subsequently, a co-culture system involving tumor cells and macrophages was established. A series of experiments, including Transwell assays, immunofluorescence staining, immunoprecipitation, flow cytometry, and immunohistochemistry, were conducted to elucidate the mechanism through which HCC cells recruit macrophages via the CCL16-CCR1 axis.

**Results:**

Single-cell analysis unveiled significant interactions between malignant HCC cells and macrophages, identifying 76 related ligand-receptor genes. Patients were classified into three subtypes based on the expression patterns of eight prognosis-related ligand-receptor genes. The subtype with the worst prognosis exhibited reduced infiltration of T cell-related immune cells, downregulation of immune checkpoint genes, and increased M2-like tumor-associated macrophage scores. *In vitro* experiments confirmed the pivotal role of the CCL16-CCR1 axis in the recruitment and M2 polarization of tumor-associated macrophages. Clinical samples demonstrated a significant association between CCL16 protein expression levels and advanced stage, lymph node metastasis, and distant metastasis. Immunohistochemistry and immunofluorescence staining further confirmed the correlation between CCL16 and CCR1, CD68, and CD206, as well as CD68+CCR1+ macrophage infiltration.

**Conclusions:**

Our study identified molecular subtypes, a prognostic model, and immune microenvironment features based on ligand-receptor interactions in malignant HCC cell communication. Moreover, we revealed the pro-tumorigenic role of HCC cells in recruiting M2-like tumor-associated macrophages through the CCL16-CCR1 axis.

## Graphical Abstract

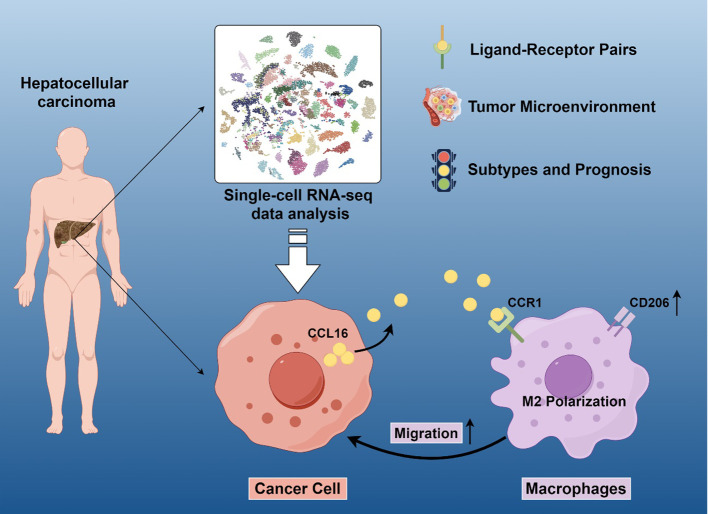

## Introduction

Hepatocellular carcinoma (HCC), a prevalent and aggressive cancer, poses a substantial global burden (1). Despite advancements in HCC diagnosis and treatment, patients with this condition continue to face an unfavorable prognosis. This underscores the urgent need for a more comprehensive understanding of the fundamental mechanisms driving tumor progression and the identification of novel treatment targets (2). The progression of HCC is profoundly influenced by the tumor microenvironment (3). This ecosystem is intricate, encompassing various cell types, including cancer cells, immune cells, stromal cells, and components of the extracellular matrix. Recognizing the diversity of the tumor microenvironment has gained increasing importance as a critical determinant of HCC prognosis (4). Unraveling the molecular mechanisms underpinning this heterogeneity and its association with patient subtyping and prognosis is essential for advancing HCC management.

Macrophages, among the diverse cell populations within the tumor microenvironment, play a pivotal role in regulating both the immune response and tumor behavior (5). In HCC, the interaction between macrophages and tumor cells holds particular significance, influencing tumor growth, invasion, metastasis, and immune evasion (6, 7). Notably, Weng et al. identified that patients with a significant presence of PPT1+ macrophages experienced heightened infiltration of CD8+ T cells and increased expression of programmed cell death-1 (PD-1) (8). Additionally, Zhao et al. demonstrated that CD168+ M2-like macrophages regulate TOP2A+CENPF+ liver cancer stem-like cells, contributing to the excessive growth of HCC (9). Furthermore, Wang et al. reported that M2 tumor-associated macrophages (TAMs) enhance stemness levels and reduce sorafenib-induced cell apoptosis through paracrine secretion of CXCL1 and CXCL2 (10). A comprehensive exploration of the intricate interaction between macrophages and cancer cells provides valuable insights into HCC development and guides the development of innovative treatment approaches (11). In this dynamic microenvironment, cell communication mediated by ligand-receptor complexes orchestrates a broad spectrum of cellular responses, significantly influencing tumor behavior (12). However, the specific ligand-receptor interactions operating within the HCC microenvironment and their functional implications remain largely unexplored.

CCL16, a chemokine renowned for its chemotactic properties, has attracted attention due to its potential involvement in HCC progression and immune modulation. While Shen et al. demonstrated its role in promoting the stemness of breast cancer cells (13), its specific implications in HCC remain unexplored. Notably, CCL16 has exhibited the ability to engage with various chemokine receptors, specifically CCR1, CCR2, CCR5, and CCR8 (14–17), expressed on immune cells, including macrophages. This interaction suggests that CCL16 could influence the tumor microenvironment by recruiting immune cells, such as macrophages, through the activation of these receptors. The presence of CCL16-related interactions may further modulate the polarization and functional properties of macrophages, potentially influencing their pro- or anti-tumorigenic activities within the HCC microenvironment. Despite these intriguing observations, the specific roles and underlying mechanisms of CCL16 in the context of HCC remain incompletely understood, warranting further dedicated investigation.

This research aimed to investigate the intricate landscape of ligand-receptor interactions within the HCC microenvironment and elucidate their functional consequences. Utilizing cutting-edge methodologies, including single-cell transcriptomic analysis and comprehensive cohort data, our objective was to characterize the complex network of ligand-receptor interactions in HCC. By employing a combination of computational analyses, experimental validations, and clinical correlations, we sought to uncover valuable insights into the role of these interactions in tumor progression and immune modulation. This comprehensive exploration aimed to contribute to our understanding of the pathogenesis of HCC, with the ultimate goal of identifying potential therapeutic targets for precision medicine approaches in the treatment of HCC.

## Methods

### Source of data and preprocessing

The count matrix for the dataset GSE149614 based on the single-cell RNA-seq technology was extracted from the GEO database (https://ncbi.nlm.nih.gov/geo/). The TCGA Liver Cancer (TCGA-LIHC) dataset’s Level 3 Bulk RNA-Seq expression data were acquired from UCSC Xena (https://xenabrowser.net/datapages/) (18), whereas the ICGC database (https://dcc.icgc.org/) provided the Level 3 data for the Liver Cancer - RIKEN, JP (LIRI-JP) dataset. For further analysis, the Bulk RNA-seq data at Level 3 underwent a log2(x+1) transformation.

### Analysis of data at the single-cell level

The single-cell data obtained from 10 liver cancer samples in GSE149614 underwent quality control and filtering with the Seurat R package (19, 20). The specific requirements were established as follows: nFeature_RNA must be between 500 and 4000, percent.mt should be less than 5, and percent.HB should be less than 1.Next, the data underwent normalization using the LogNormalize technique via the NormalizeData function. The FindVariableFeatures function was used to identify 2000 genes that exhibit high variability in each sample. Afterwards, the CCA method was utilized to eliminate batch discrepancies and merge the information. Principal Component Analysis was performed using the RunPCA function, with the parameters dims set to 20 and resolution set to 0.5. The identification of cell clusters was done using the FindNeighbors and FindClusters functions. The FindMarkers function was used to differentiate cell populations and identify marker genes for each cluster. Using default parameters, the CopyKAT R package was utilized to estimate Copy Number Variations (CNV) for every cell, enabling the detection of aneuploid cells (21). In the end, we utilized the CellChat R package (22) to deduce and examine the cellular communication network and discover ligand-receptor pairs linked to aneuploid cells.

### Identification of ligand-receptor-related subtypes

To further select prognostic-related variables, univariate Cox regression analysis was conducted in the TCGA-LIHC cohort, based on the expression profiles of ligand-receptor genes linked to aneuploid cells. Afterwards, the ConsensusClusterPlus R package (23) was applied to perform separate unsupervised clustering in the TCGA-LIH (N = 365) and LIRI-JP (N = 232) cohorts. The clusterAlg parameter utilized was pam, with the Euclidean distance, 500 reps, a pItem of 0.8, and pFeature of 1. The log-rank test from the survival package was utilized to conduct survival analysis among various subtypes, resulting in the generation of Kaplan-Meier curves.

### Analysis of the tumor microenvironment using quantitative methods

Using the GSVA package, we assessed the levels of 28 immune cell types’ infiltration in the TCGA-LIHC cohort through the single-sample Gene Set Enrichment Analysis (ssGSEA) technique. The background gene set was referenced from the previous study (24). The Stromal score and Immune Score for each sample were calculated using the Estimate R package. We examined the variation in expression of immune checkpoint genes among different groups by utilizing a gene list obtained from a prior study (25). Afterwards, the gene expression patterns were normalized using the scale method, and the Tumor Immune Dysfunction and Exclusion (TIDE) score for each specimen was calculated using the TIDE online tool (http://tide.dfci.harvard.edu/) (26, 27). The ssGSEA method mentioned earlier was used for quantification of all gene sets in this study, and Wilcoxon test was applied for differential analysis.

### Detection of variably expressed genes across subtypes

Using the eBayes test method from the limma R package, we performed a comparative analysis of gene expression profiles across various subtypes. For each subtype, genes that exhibited a |FoldChange| > 1.5 and fdr < 0.05 were identified as differentially expressed. Afterwards, the clusterProfiler R package was utilized to perform functional enrichment analysis (28).

### Construction of the prognostic model and nomogram

We initially conducted univariate Cox analysis to select initial variables with a threshold of P < 0.05, based on the distinct genes among various molecular subtypes. Afterwards, we employed the glmnet R software package to conduct Lasso regression analysis, specifically in the TCGA-LIHC group. To identify variables corresponding to lambda.min, Cox regression was performed using 10-fold cross-validation. Next, a stepwise regression analysis was utilized to construct the model for computing the risk score of every individual. Validation of the identical model was conducted in the LIRI-JP group. Patients were classified into high- and low-risk groups based on the cohort’s median risk score as the threshold. The survival package was utilized for conducting survival analysis, while the timeROC R package was employed to generate receiver operating characteristic (ROC) curves for assessing the predictive performance based on the Area Under Curve (AUC). Furthermore, the Wilcoxon test was employed to examine the disparities in the immune microenvironment among the two cohorts, while the Pearson correlation test was utilized to explore the association between the risk score and both the immune score and TIDE score. Prognostic impact of the risk score and clinical variables was observed in the TCGA-LIHC cohort through univariate and multivariate Cox analyses. The rms R package was utilized to construct a nomogram by integrating important variables. Afterwards, AUC curves were generated by utilizing the plotAUCcurve function from the timeROC R package, calibration curves were produced using the calibrate function, and the decision curve was plotted with the assistance of the rmda R package.

### Cell culture

The Chinese Academy of Sciences Cell Bank provided HEK293T cells and liver cancer cell lines HEPG2/JHH7/HUH7/SNU761. DMEM medium supplemented with 10% fetal bovine serum (FBS) was used to culture HEPG2 and HUH7 cells, whereas RPMI1640 medium supplemented with 10% FBS was used to culture JHH7 and SNU761 cells. The THP1 cells, acquired from our lab, were grown in RPMI1640 solution with 10% fetal bovine serum that had been heat-inactivated, along with 10 U/mL penicillin and 10μg/mL streptomycin. The cells were cultured in a flask with a cell density of around 1×10^8^, in an incubator at a temperature of 37°C and with a CO_2_ concentration of 5%. Cultured THP-1 cells were thinned to a concentration of 1 million cells per milliliter and then placed in culture dishes with a diameter of 35 millimeters. Afterwards, the cells were prompted to undergo differentiation by being incubated in RPMI1640 solution with a concentration of 100 ng/ml of phorbol 12-myristate 13-acetate (PMA) for a duration of 48 hours. Summary of relevant reagent information in the experimental section of this study can be found in **Table 1**.

**Table 1:** Reagent information used in this study.

Reagent Name	Brand	Catalog Number
anti-CCL16	Abcam	Ab199162
Human CCL16 ELISA kit	Abcam	Ab243673
anti-CCR1	Invitrogen	PA1-41062
anti-CCR2	CST	D14H7
anti-CCR5	Invitrogen	PA5-85136
anti-CCR8	Abcam	Ab32399
anti-CD68	Abcam	Ab213363
anti-CD206	Abcam	Ab64693
anti-β-actin	Sigma-Aldrich	A2228
anti-Flag	Sigma-Aldrich	A8592
Viability Dye eFluor™ 780	eBioscience	65-0865-18
CD68-FITC	Biolegend	333806
CD80-FITC	Biolegend	305206
CD206-PE Cyanine7	Biolegend	321124
Age	NEB	R3552L
EcoR I	NEB	R3101L
Goat anti-Mouse IgG (H+L) Secondary Antibody, Alexa Fluor Plus 555	Thermo Fisher	A21424
Goat anti-Rabbit IgG (H+L) Secondary Antibody, Alexa Fluor Plus 488	Thermo Fisher	A11034
Protein A/G magnetic beads	MedChemExpress	HY-K0202
LipoFiterTM Liposomal Transfection Reagent	Hanbio	HB-LF10001
Puromycin	Sangon Biotech	A610593
BSA	Sangon Biotech	A602440
PMA	Sigma	p1585-1MG
Recombinant flag-CCL16	Novoprotein	C064
BX471	MedChemExpress	HY-12080

### Cell line knockdown and overexpression

To generate HEPG2 cells with CCL16 knockdown and THP1 cells with CCR1 knockdown, validated shRNA sequences targeting the respective proteins were searched online at the Sigma-Aldrich website (https://www.sigmainformatics.com/Informatics_tools/batch-search.php). Primers were designed based on the selected sequences and cloned into the plKO.1 vector. Subsequently, the plKO.1 vector was co-transfected with the lentiviral packaging vectors psPAX2 and pMD2 into HEK293T cells. After 48 hours, the viral supernatant was collected and used to infect the target cell lines. The infected cells were then selected using 4μg/mL puromycin, and the knockdown efficiency of the target proteins was assessed by qPCR. The shRNA sequences for CCL16 knockdown were as follows: shCCL16-1: TCCTTATCATTACTTCGGCTT, shCCL16-2: AGGAGAAGTATTTCGAATATT. The shRNA sequences for CCR1 knockdown were as follows: shCCR1-1: CCCTACAATTTGACTATACTT, shCCR1-2: CCCTGGTAGAAAGAAGATGAA. To generate SNU761 cells overexpressing CCL16, a Plvx-IRES-ZsGreen1-flag-CCL16 overexpression plasmid was constructed. Subsequently, the Plvx-IRES-ZsGreen1 vector was co-transfected with the lentiviral packaging vectors psPAX2 and pMD2 into HEK293T cells. After 48 hours, the viral supernatant was collected and used to infect SNU761 cells. The infected cells were then selected using 4μg/mL puromycin, and the efficiency of CCL16 overexpression was assessed by qPCR.

### Macrophages migration and co-culture assays using Transwell

For the migration assay of macrophages, a 24-well Transwell chamber system with an 8μm membrane was used. PMA-induced THP1 cells (5×10^4^ cells/well) were placed in the upper chamber, while the conditioned medium from the control, CCL16-knockdown HEPG2 cells/CCL16-overexpressing SNU761 cells was placed in the lower chamber. Following a 24-hour period, the THP1 cells that moved towards the bottom chamber were dyed using 0.1% crystal violet. To calculate the average, three fields were randomly chosen under a microscope to determine the number of migrated cells. In the co-culture assay of macrophages and liver cancer cells, a 6-well Transwell chamber system with a 0.4μm membrane was used to separate the upper and lower chambers. Control, CCL16-knockdown HEPG2 cells/CCL16-overexpressing SNU761 cells (1×10^5^ cells/well) were placed in the lower chamber, while PMA-induced THP1 cells (1×10^5^ cells/well) were placed in the upper chamber. After 48 hours of co-culture, the THP1 cells in the upper chamber were subjected to flow cytometry. Three independent replicates were conducted.

### Elisa

CCL16 expression in the control, CCL16-knockdown HEPG2 cells, and CCL16-overexpressing SNU761 cells were measured using human CCL16 ELISA kits (Abcam). The particular experimental protocols were carried out in accordance with the provided guidelines. Three independent replicates were conducted.

### Immunofluorescence

The SNU761 cells overexpressing CCL16 were co-cultured with PMA-treated THP1 cells. Afterward, the cells were fixed with 4% paraformaldehyde for 10 minutes, blocked with PBS containing 2% BSA for 30 minutes, and then incubated overnight at 4°C with antibodies against Flag and CCR1. On the following day, the cells were stained with corresponding fluorescent secondary antibodies for 1 hour at room temperature. The cell nuclei were stained with DAPI. Afterward, the slides were mounted using an anti-fade mounting medium and observed under a ZEISS microscope for fluorescence imaging. Liver cancer tissue samples were embedded in paraffin and prepared as sections. The sections underwent a series of steps including deparaffinization, hydration, and antigen retrieval. After blocking with 5% goat serum, the sections were incubated overnight at 4°C with appropriately diluted CD68 and CCR1 antibodies. On the next day, the sections were stained with corresponding fluorescent secondary antibodies for 1 hour at room temperature. Three distinct fields were randomly selected from each slide, and the number of CD68+CCR1+ cells was quantified. Three independent replicates were conducted. The average value of the cell counts from the three fields was used for statistical analysis.

### Coimmunoprecipitation

Flag-CCL16 recombinant protein was added into THP1 cells. Following a 24-hour period, the cells were disrupted by employing RIPA buffer that consisted of proteinase inhibitors. Ten percent of the entire cell lysate was retained as the control sample, whereas the rest of the lysate was subjected to an overnight incubation at 4°C with Flag and IgG antibody. On the following day, beads containing protein A/G were introduced and left to incubate for an extra 4 hours. Afterwards, the beads were rinsed thrice using RIPA solution and heated in 20μL of 1x loading buffer. Flag/CCR1/CCR2/CCR5/CCR8 antibodies were utilized for immunoblotting analysis to identify the interacting proteins, in addition to the input protein samples. Three independent replicates were conducted.

### Western blot

After undergoing SDS-PAGE gel electrophoresis, the proteins were then transferred onto a nitrocellulose (NC) membrane. Specific primary antibodies were incubated with the membrane overnight at 4°C. Following this, the sample was exposed to secondary antibodies for a duration of 1 hour at room temperature. The Immobilon Western chemiluminescent HRP substrate (Millipore) was utilized to visualize protein bands on a luminescent imaging workstation (Tanon). ImageJ software was used to measure the band intensities of various proteins, with ACTIN serving as the chosen loading control.

### qPCR

TRIzol reagent was used to extract total RNA according to the instructions provided by the manufacturer. The PrimeScript RT Reagent Kit (Takara) was utilized for the synthesis of cDNA. The SYBR Green Master mix (Roche) was utilized to perform quantitative real-time PCR (QPCR). The 2^-ΔΔCt^ method was utilized to determine the comparative levels of gene expression, which were then normalized to the expression of ACTIN. **Table 2** contains a list of the specific primers utilized for gene-specific PCR.

**Table 2:** Primer sequences for qPCR.

Symbol	Fwd	Rvs
CCL16	CTTATCATTACTTCGGCTTCTCGC	GGCCTTTCTGTATCCCACCACTA
CCR1	CCTGCTGACGATTGACAGGT	AGGGCCCAAATGATGATGCT
ACTIN	GTCTCCTCTGACTTCAACAGCG	CGTACAGGTCTTTGCGGATG

### Flow cytometry

After 48 hours of co-culture between macrophages and liver cancer cells in a 0.4μm pore size Transwell system, the PMA induced THP1 cells from the upper chamber were subjected to flow cytometry analysis. Cells were incubated with CD80-FITC and CD206-PE Cyanine7 flow cytometry antibodies at 4°C for 30 minutes. Following the addition of 1 mL PBS and centrifugation at 1500 rpm for 5 minutes, the cell pellet was resuspended in 200μL PBS. Flow cytometry analysis was performed using the Beckman Gallios flow cytometer, and the experimental results were analyzed using Flow Jo. Three independent replicates were conducted.

### Immunohistochemistry

Liver cancer tissues were embedded in paraffin and then cut into sections. After deparaffinization and hydration, the sections were subjected to antigen retrieval. The activity of endogenous peroxidase was inhibited using 3% H_2_O_2_, followed by blocking the sections with 5% goat serum. Antibodies against CCL16, CD68, and CD206 were diluted, added, and incubated overnight at 4°C. Following the PBST (PBS with Tween 20) wash, the sections were exposed to biotinylated secondary antibodies and left at ambient temperature for a duration of 20 minutes. After being washed three times with PBST, the sections were then exposed to streptavidin-horseradish peroxidase at room temperature for a duration of 15 minutes. After three washes with PBST, fresh DAB working solution was added for color development. To halt the reaction, the sections were rinsed with water for a duration of 5 minutes, and subsequently examined under a ZEISS microscope. The Image Pro Plus software was used to calculate the Mean Optical Density (MOD) value.

### Ethics statement

The research was carried out with the endorsement of the Ethics Committee at the First Hospital of China Medical University. All participants provided written consent prior to the study.

### Statistical analysis

GraphPad Prism 8 was utilized for conducting statistical analysis. Means ± standard deviations (SD) were used to present quantitative data, whereas numbers were used to present qualitative values. For the analysis of suitable quantitative data, either the Unpaired Student’s t-test or two-way ANOVA was utilized. Statistical significance was determined by a two-sided *P*-value less than 0.05, indicated as * for *P* < 0.05, ** for *P* < 0.01, and *** for *P* < 0.001.

## Results

### The microenvironment of malignant liver cancer cells exhibits cellular communication characteristics, with macrophages showing the most notable interaction

**Figure 1:**
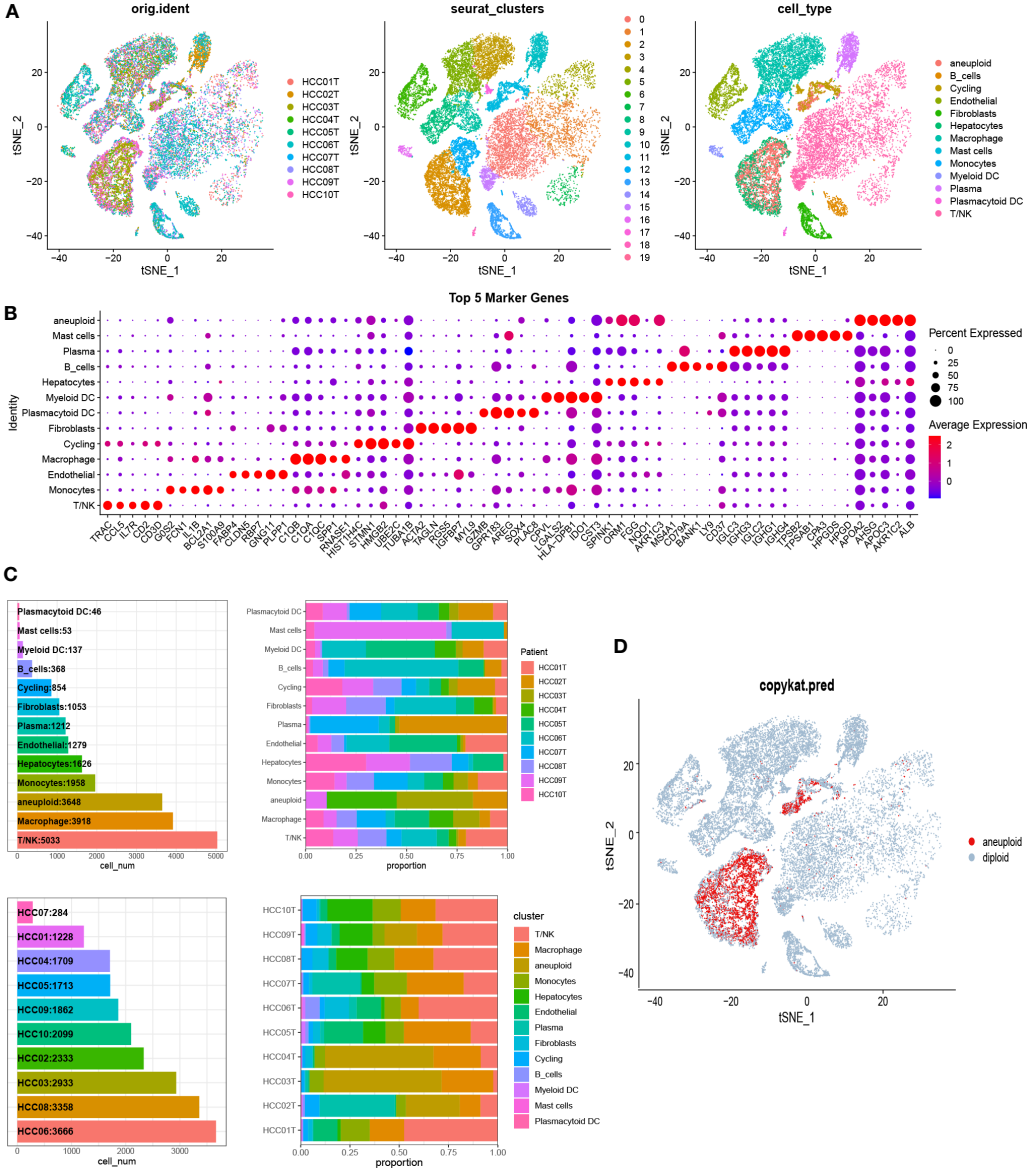
Single-cell analysis identified cellular subpopulation categories and compositional features of liver cancer tissue samples. **(A)** The t-SNE clustering plot was generated after quality control and integration using the canonical correlation analysis (CCA) method. The left panel shows the t-SNE clustering of 10 HCC patients. The middle panel represents cell subgroups identified using the Seurat R package’s FindClusters function with a resolution of 0.5. The right panel displays the t-SNE clustering of cell subpopulations after manual annotation. **(B)** Based on the ascending order of Wilcoxon test values, the top 5 marker genes for each cell type were identified using the FindMarkers function. **(C)** The number of cells for each cell type and the cellular composition of each sample. **(D)** The t-SNE distribution of malignant aneuploid cells identified by CopyKAT.

**Figure 2:**
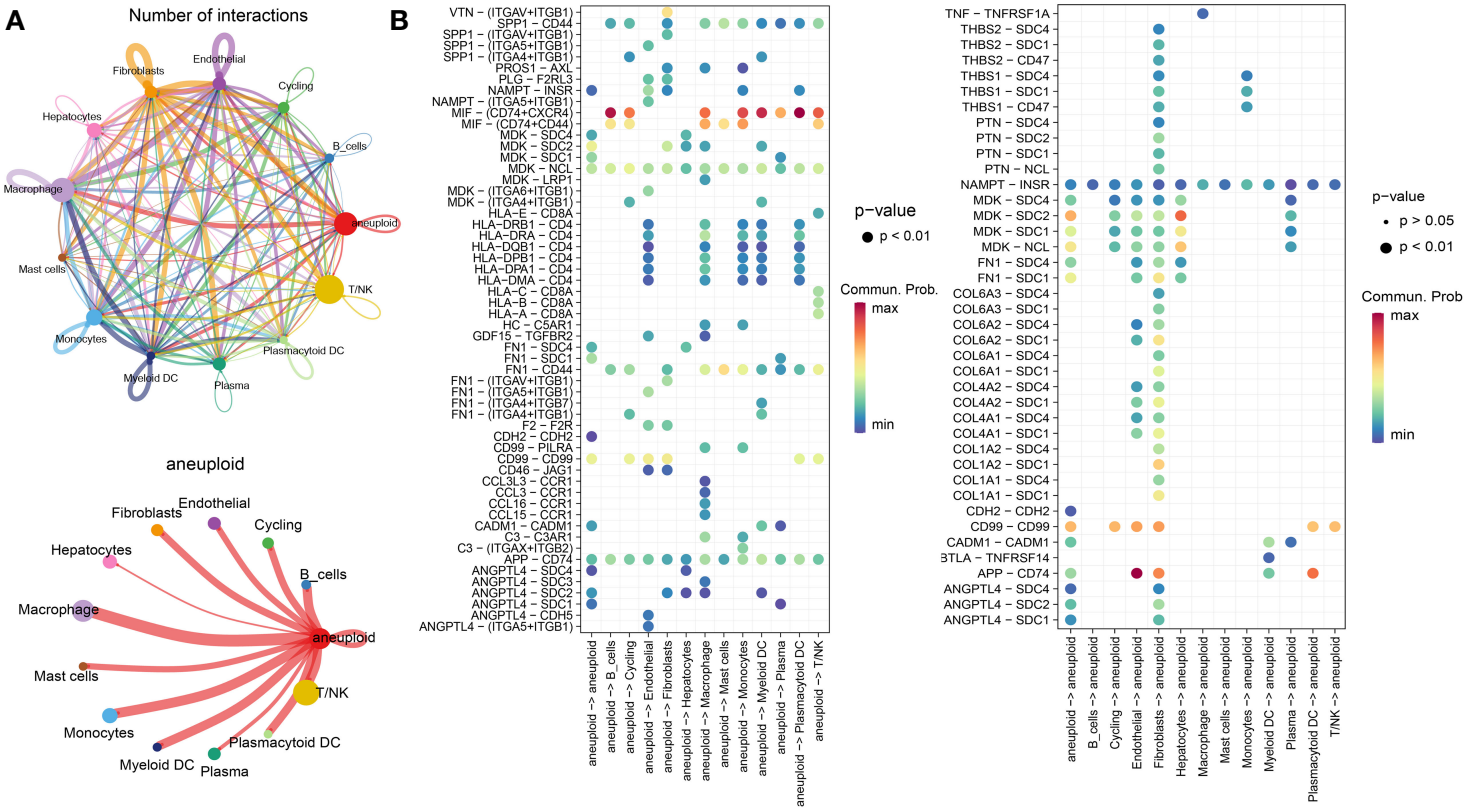
Cellular communication features of malignant liver cancer cells in the microenvironment. **(A)** The number (top) and weight (bottom) of interactions between different cell types as identified by CellChat analysis. **(B)** Enrichment results of receptor-ligand pairs, with aneuploid cells as the source (left) and receptor cells (right), as determined by CellChat.

To examine the communication properties of cancerous liver cells within the tumor microenvironment, we conducted an analysis of single-cell transcriptomic data using tissue samples obtained from 10 individuals diagnosed with malignant liver cancer. After quality control and clustering, we obtained a total of 13 cell types, comprising 44,320 cells (**Figure 1A**), including 3,648 aneuploid cells. In addition, we showcased the leading 5 marker genes for every cell cluster (**Figure 1B**). Among all cell types, T/NK cells had the highest proportion, followed by Macrophages, aneuploid cells, and Monocytes. On the other hand, there was significant heterogeneity in the composition of cell types between different patients (**Figure 1C**). The tSNE distribution of aneuploid cells identified by CopyKAT primarily overlapped with Hepatocytes and Cycling cells, demonstrating high accuracy (**Figure 1D**). The interactions between cell clusters identified by Cellchat revealed that the most prominent interacting cell types with aneuploid cells were macrophages and monocytes, followed by fibroblasts and myeloid DC cells (**Figure 2A**). Cell-cell interactions are often mediated by receptor-ligand pairs, with aneuploid cells acting as either source or receptor cells. Through Cellchat enrichment analysis, we identified 96 pairs of important receptor-ligand pairs, with a significant enrichment of aneuploid cell-derived interactions with other cell types compared to their interactions as receptor cells (**Supplementary File 1**). Particularly, the potential receptor-ligand pairs involving aneuploid cells acting on macrophages were found to be at a higher level compared to other cell types, including the CCL16-CCR1 axis (**Figure 2B**). These findings suggest that cell-cell interactions initiated by aneuploid cells as source cells are crucial role in the immune microenvironment of liver cancer.

### Molecular subtyping and immune cell infiltration characteristics based on prognostic receptor-ligand genes

**Figure 3:**
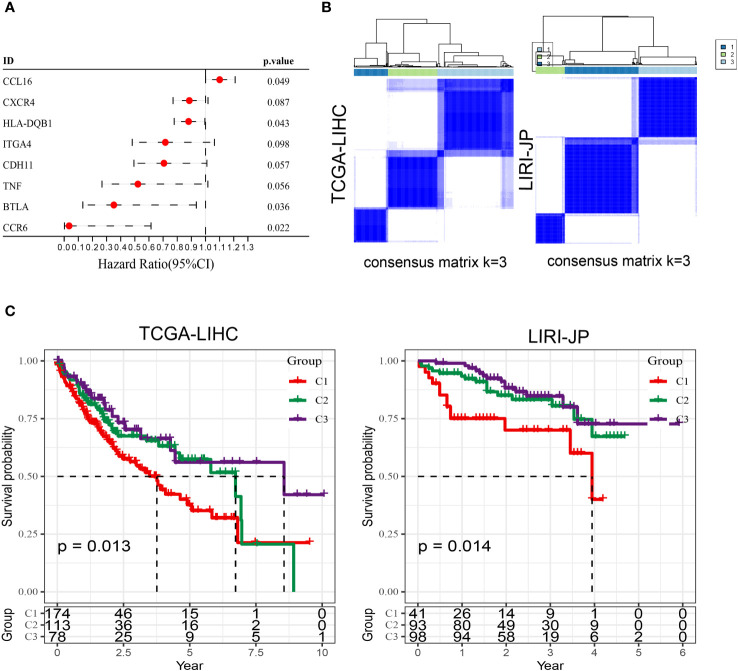
Molecular subtyping based on prognostic receptor-ligand genes in TCGA-LIHC and LIRI-JP cohorts. **(A)** Univariate Cox regression analysis results of receptor-ligand genes in the TCGA-LIHC cohort. **(B)** Unsupervised clustering of the two cohorts based on the expression pattern of prognosis-associated receptor-ligand genes. **(C)** Kaplan-Meier survival curves of the three subtypes in both cohorts. Log-rank test.

As is well-established, tumor cells exhibit pronounced heterogeneity, and similarly, the intercellular interactions based on aneuploid cells are also expected to display such complexity (29). Therefore, leveraging the previously identified core set of receptor-ligand genes (comprising a total of 76 genes) associated with aneuploid cells, we conducted an extensive investigation of prognosis and molecular subtyping in two large-scale cohorts, namely TCGA-LIHC (training cohort) and LIRI-JP (testing cohort). Using a strict univariate Cox regression analysis with a p-value threshold of less than 0.1, we were able to identify 8 receptor-ligand genes that have significant prognostic value in the TCGA-LIHC cohort. Significantly, CCL16 stood out as the only factor posing a risk among these candidates (**Figure 3A**). Using the expression patterns of these eight genes, patients from both cohorts were successfully classified into three distinct subtypes through unsupervised clustering analysis (**Figure 3B**). Significantly, all three subcategories demonstrated comparable prognostic disparities in both datasets, underscoring the strength and uniformity of the categorization (**Figure 3C**). The analysis of immune cell infiltration indicated that the C1 subtype, which had the worst prognosis, displayed notably reduced scores for the majority of immune cell categories, specifically T cells, including Activated CD4 T, Central memory CD4 T, and Central memory CD8 T, in contrast to the C2 and C3 subtypes (**Supplementary Figure 1A**). Consistently, the results of the Estimate analysis also indicated a notable decrease in the Stromal score and Immune score within the C1 subtype (**Supplementary Figure 1B**). Moreover, the levels of gene expression for most immune checkpoint genes were notably decreased in the C1 subtype when compared to the other two subtypes, indicating a possible diminished immune response in the C1 subtype (**Supplementary Figure 1C**). According to the TIDE analysis, it was found that the C1 subtype exhibited notably reduced scores in relation to the IFNG pathway, which is linked to the innate immune responses (30). Remarkably, in circumstances of generally limited immune cell penetration, the C1 category, associated with the most unfavorable prognosis, exhibited notably increased ratings for M2 macrophages linked to tumors (**Supplementary Figure 1D**). Therefore, we speculate that the polarization of macrophages towards M2 phenotype might have a crucial impact on the progression of liver cancer.

### Construction of the four-gene risk model and prognostic nomogram

**Figure 4:**
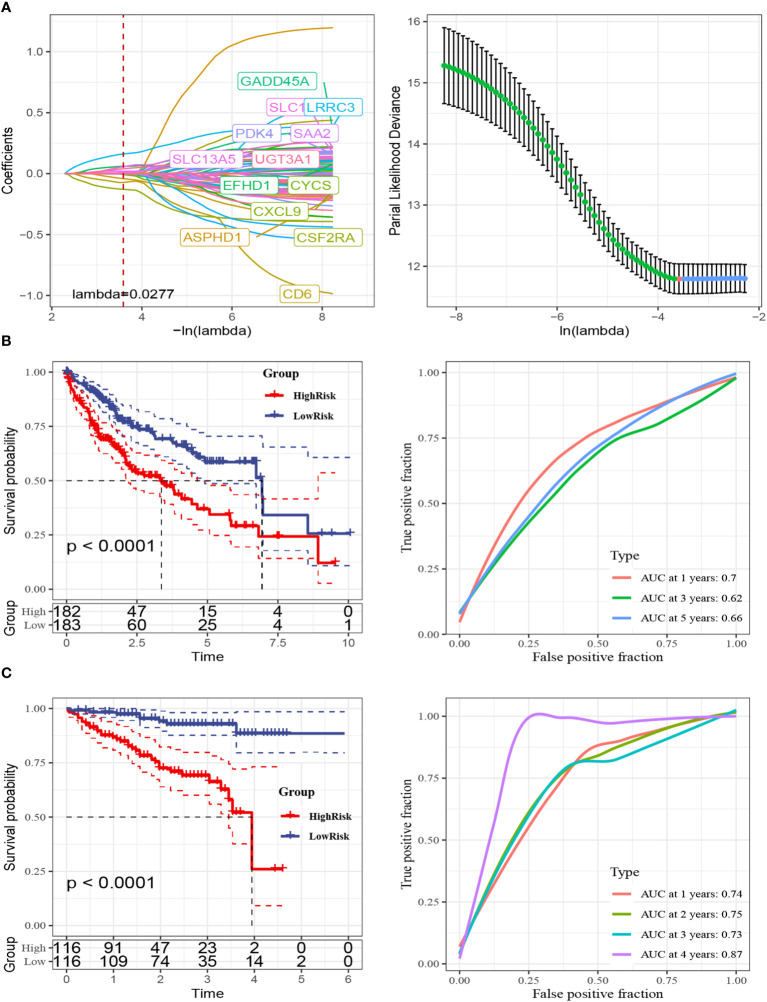
Construction of the 4-gene risk model. **(A)** Lasso regression analysis was performed to assess model fit. The analysis included two components. Left: The curve of partial-likelihood deviance was plotted against Log(λ), where a smaller value indicated a better fit of the model. Right: The curve of regression coefficients for each variable was plotted against the change of Log(λ). **(B)** Kaplan-Meier survival curves and 1, 3, 5-year ROC curves were generated for the high-risk and low-risk groups in the TCGA-LIHC cohort. **(C)** Kaplan-Meier survival curves and 1, 3, 5-year ROC curves were generated for the high-risk and low-risk groups in the LIRI-JP cohort. The Log-Rank test was used for statistical analysis.

**Figure 5:**
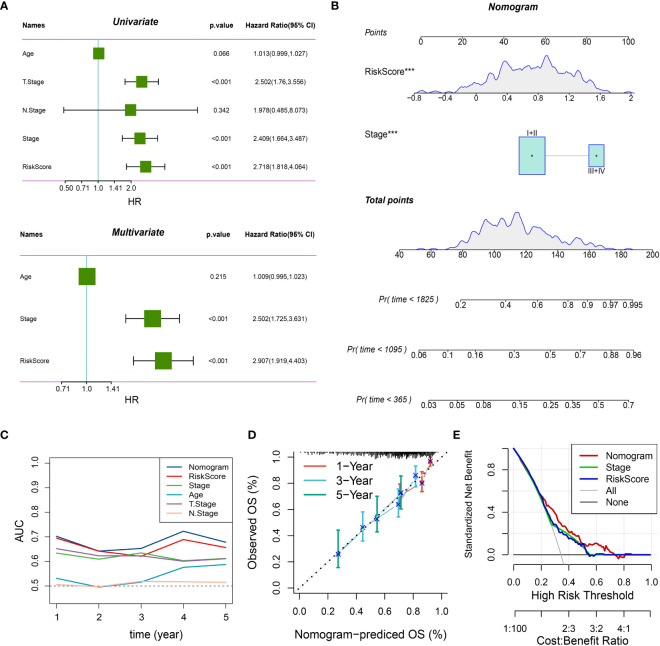
Integrated nomogram with improved predictive performance. **(A)** Results of univariate and multivariate analyses of risk score and clinical variables in the TCGA-LIHC cohort. P < 0.05 indicates statistical significance. **(B)** Integrated prognostic nomogram combining risk score and stage for predicting 1, 3, and 5-year patient outcomes. ***: P < 0.001 in the multivariate Cox regression analysis. **(C)** AUC curves of the nomogram and other variables, showing the highest accuracy of the nomogram across different survival time points. **(D)** Calibration curves of the nomogram, with closer proximity to the diagonal line indicating a closer match between predicted and actual event rates. **(E)** Decision curve analysis (DCA) curves for different variables, demonstrating the highest standard net benefit of the nomogram.

Afterwards, we conducted differential gene expression analysis on the three subtypes. The genes that showed differential expression were analyzed for functional enrichment (**Supplementary Figure 2A, B**; **Supplementary File 2**) and used to construct a prognostic model. Initially, we conducted univariate Cox analysis on these genes that were expressed differentially (DEGs) in the TCGA-LIHC cohort, employing a threshold of *P* < 0.05. This yielded 274 prognostic-related DEGs. Following that, an analysis using Lasso regression was performed, leading to the identification of 13 genes (**Figure 4A**). By employing a stepwise regression approach, we eventually pinpointed four genes and developed a risk model to calculate the risk score with the following formula: risk score = -0.349 * *CSF2RA* + 0.245 * *LRRC3 +* 0.095 * *UGT3A1 +* 0.115 * *EFHD1*. Risk scores were computed for every patient in both the TCGA-LIHC and LIRI-JP cohorts (**Supplementary File 3**). Patients were categorized into high and low-risk groups based on the threshold set by the median value. The findings indicated that the high-risk category exhibited notably worse prognosis. In addition, the AUC plots demonstrated good predictive precision (**Figures 4B, C**). In order to improve the accuracy of predictions, we took into account the inclusion of clinical factors in the model. In the multivariable Cox analysis (**Figure 5A**), it was found that the risk score and stage continued to be significant prognostic factors, demonstrating their independence. Afterwards, we built a comprehensive calibration graph (**Figure 5B**). According to **Figure 5C**, the AUC curve indicated that the nomogram outperformed other clinical factors like the risk score and stage in terms of predictive accuracy. Moreover, the calibration plot demonstrated a strong correlation between the estimated and observed occurrence frequencies (**Figure 5D**), while the decision curve analysis (DCA) graph displayed the greatest overall net advantage for the nomogram (**Figure 5E**). In addition, a substantial inverse relationship was noted between the risk score and immune score (R = -0.23, P = 6.4e-6) (**Supplementary Figure 3A**). The group with minimal risk displayed notably elevated levels of Tumor-Reactive T Cell Signature (TRS score), which measures the responsiveness of T cells towards tumors (31), and increased levels of cytolytic activity score (CYT score), which evaluates the effectiveness of T cells in targeting tumor cells within the tumor microenvironment (32). Nevertheless, no notable distinction was detected between the two cohorts regarding the Th1/IFNγ genetic pattern (**Supplementary Figure 3B**). In contrast to the high-risk category, the low-risk category demonstrated elevated PD-1 and CTLA4 expression, along with a notable rise in the infiltration of immune cells related to T cells (**Supplementary Figure 3C, D**).

### Tumor cell-derived CCL16 mediates the recruitment and M2 polarization of macrophages in the liver cancer microenvironment

**Figure 6:**
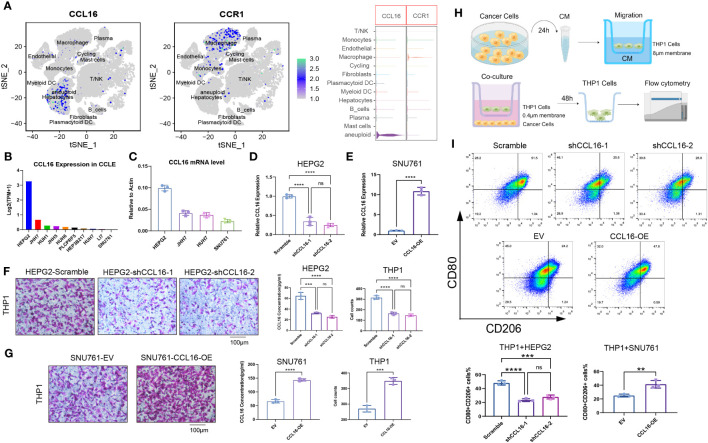
Tumor cell-derived CCL16 mediates the recruitment and M2 polarization of macrophages in the liver cancer microenvironment. **(A)** Expression of CCL16 and CCR1 in different cell types at the single-cell level. **(B)** Expression levels of CCL16 in different liver cancer cell lines from the CCLE database. **(C)** mRNA expression of CCL16 in different liver cancer cell lines detected by qPCR. **(D)** Validation of CCL16 knockdown in HEPG2 cell line. **(E)** Validation of CCL16 overexpression in SNU761 cell line. **(F)** Transwell assay to evaluate the recruitment of THP1 cells by control and CCL16 knockdown HEPG2 cells, and ELISA assay to measure CCL16 concentration in the culture supernatant of HEPG2 cells. **(G)** Transwell assay to evaluate the recruitment of THP1 cells by control and CCL16 overexpressing SNU761 cells, and ELISA assay to measure CCL16 concentration in the culture supernatant of SNU761 cells. **(H)** Schematic diagram illustrating the cell co-culture system and the macrophage migration assay. **(I)** Flow cytometry analysis to detect the proportion of M2-polarized cells after co-culture of THP1 cells with CCL16 knockdown or overexpressing tumor cells. Transwell Scale Bar = 100μm. Three independent replicates were conducted. Statistical data are presented as mean ± SD, and each data point represents an independent measurement. Unpaired Student’s t-test. ns, not significant; **: *P* < 0.01; ***: *P* < 0.001; ****: *P* < 0.0001.

Through single-cell data analysis, we identified specific expression of the chemokine CCL16 in liver cancer cells, while its receptor CCR1 was specifically expressed in macrophages (**Figure 6A**). Our hypothesis suggests that the heightened occurrence of M2 macrophages in the microenvironment of liver cancer could potentially be attributed to CCL16-CCR1. In order to examine this hypothesis, we initially examined the CCL16 expression in various liver cancer cell lines by utilizing the CCLE database (**Figure 6B**). Subsequently, we validated the mRNA expressions of CCL16 in several liver cancer cell lines available in our laboratory through qPCR, which were consistent with the database results, with HEPG2 cells showing the highest expression and SNU761 cells showing the lowest expression (**Figure 6C**). Next, to confirm whether CCL16 expressed by liver cancer cells is associated with macrophage recruitment, we generated HEPG2 cells with CCL16 knockdown and SNU761 cells with CCL16 overexpression (**Figures 6D, E**). Transwell experiments revealed that knocking down CCL16 expression in HEPG2 cells significantly reduced their recruitment of THP1 cells, while overexpressing CCL16 promoted the recruitment of THP1 cells by SNU761 cells (**Figures 6F, G**). Given the widespread existence of tumor-associated macrophages (TAMs) in the tumor microenvironment, known for their M2-polarized characteristics linked to tumor advancement, we utilized a flow cytometry technique (33) that was previously explained to assess the polarization condition of macrophages in the co-culture setup. The schematic diagram illustrating the cell co-culture system and the macrophage migration assay is presented (**Figure 6H**). M1 and M2 macrophages were distinguished using CD80 as a common marker, whereas CD206 was exclusively used as a marker for M2 macrophages (34). The findings indicated that the combination of THP1 cells with HEPG2 cells lacking CCL16 expression notably decreased the percentage of CD80+CD206+ cells in THP1.In contrast, co-culturing THP1 cells with SNU761 cells overexpressing CCL16 significantly increased the proportion of CD80+CD206+ cells in THP1 (**Figure 6I**). The findings present convincing proof that cancer cells attract macrophages and support their M2 polarization by releasing CCL16.

### The recruitment of tumor-associated macrophages mediated by CCL16 depends on the macrophage receptor CCR1

**Figure 7:**
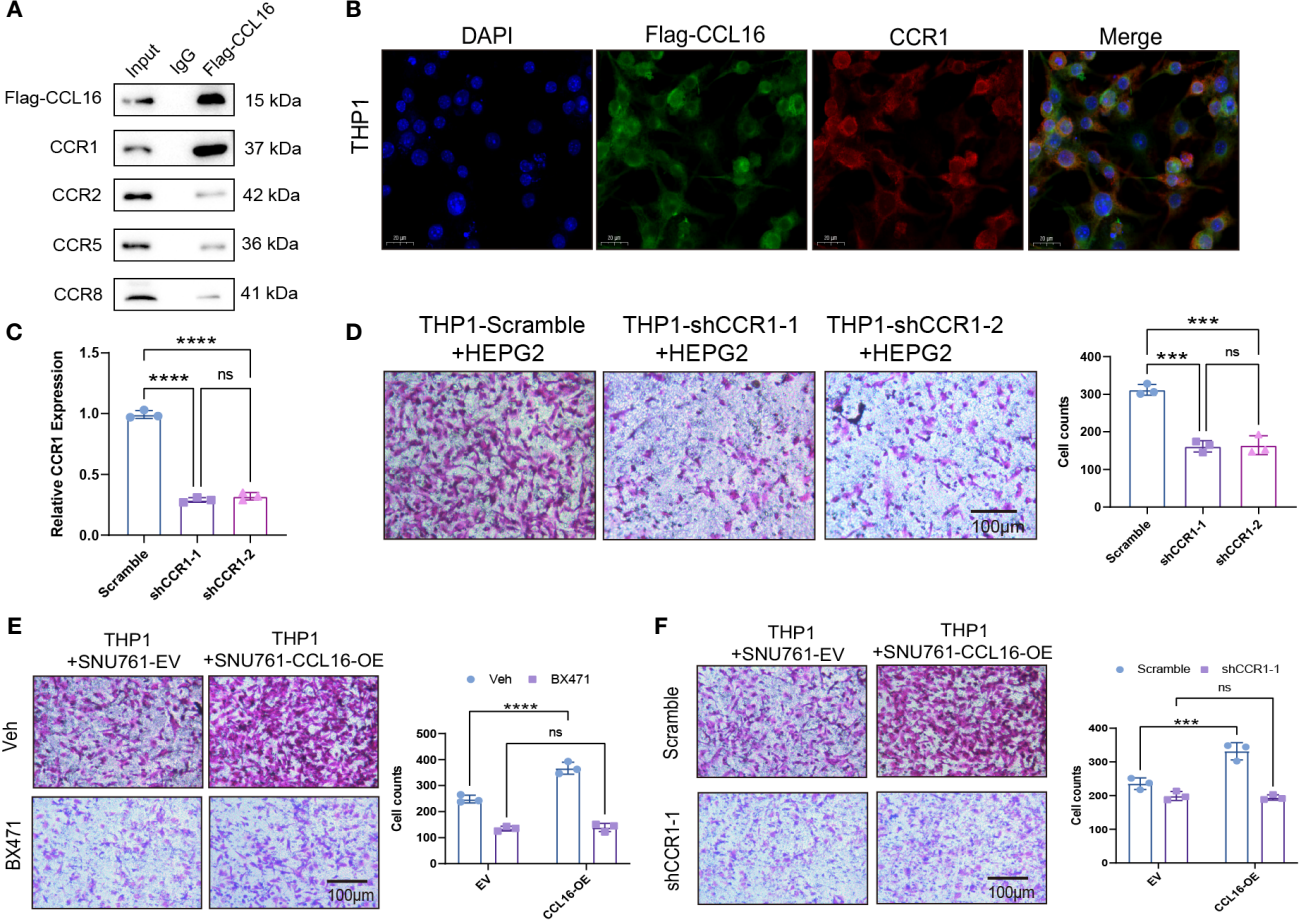
The recruitment of tumor-associated macrophages mediated by CCL16 depends on the macrophage receptor CCR1. **(A)** Immunoprecipitation assay to detect the interaction between Flag-CCL16 and CCR1, CCR2, CCR5, CCR8 in THP1 cell culture medium. **(B)** Immunofluorescence assay to detect the co-localization of Flag-CCL16 and CCR1 in THP1 cells after the addition of Flag-CCL16. Scale bar = 20 μm. **(C)** qPCR validation of CCR1 knockdown in THP1 cells. **(D)** Transwell assay to evaluate the cell migration ability of CCR1 knockdown THP1 cells co-cultured with HEPG2 cells. **(E)** Recruitment of THP1 cells by control or 5μM BX471-treated CCL16 overexpressing SNU761 cells after 24 hours. **(F)** Recruitment of THP1 cells by CCL16 overexpressing SNU761 cells after CCR1 knockdown. Transwell Scale Bar = 100μm. Three independent replicates were conducted. Statistical data are presented as mean ± SD, and each data point represents an independent measurement. Unpaired Student’s t-test or two-way ANOVA was used for statistical analysis. ns, not significant; ***: *P* < 0.001; ****: *P* < 0.0001.

Next, we aimed to identify the receptor through which CCL16 acts on macrophages. There have been studies reporting that CCL16 binds to known receptors including CCR1, CCR2, CCR5, and CCR8 (14–17). We added synthetic Flag-CCL16 protein into THP1 cells cultured *in vitro* and performed co-immunoprecipitation experiments. The results revealed that CCL16 predominantly interacts with the CCR1 receptor on macrophages, while the binding affinity to other receptors was minimal (**Figure 7A**). This interaction was further confirmed by immunofluorescence, showing colocalization of CCL16 and CCR1 on the cell membrane of THP1 cells (**Figure 7B**). To further investigate the role of CCR1, we performed CCR1 knockdown in THP1 cells (**Figure 7C**) and co-cultured them with HEPG2 cells to assess the migration ability of macrophages. The results demonstrated that CCR1 knockdown in THP1 cells significantly inhibited the recruitment of macrophages by HEPG2 cells (**Figure 7D**). Interestingly, after treatment with the CCR1 inhibitor BX471, the overexpression of CCL16 no longer promoted THP1 cell recruitment (**Figure 7E**). Consistently, when CCR1 was knocked down in THP1 cells, co-culture with tumor cells overexpressing CCL16 no longer facilitated macrophage recruitment (**Figure 7F**). These results demonstrate that the secretion of CCL16 by liver cancer cells promotes macrophage recruitment through binding to the CCR1 receptor on macrophages.

### M2 macrophage infiltration and CCR1 expression in clinical tissues are positively correlated with CCL16

**Figure 8:**
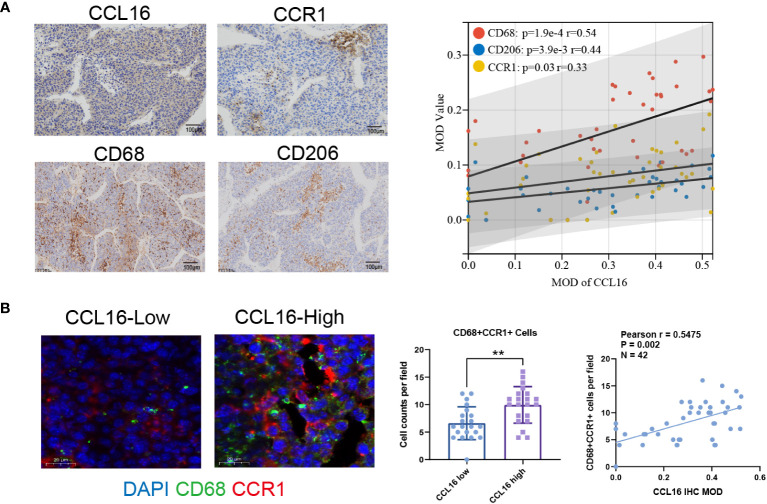
High expression of CCL16 is positively correlated with the infiltration of M2 macrophages and the expression of CCR1 in clinical tissues. **(A)** Immunohistochemistry representative images and Pearson correlation analysis of CCL16 with CCR1, CD68, and CD206 in liver cancer patient samples, as well as the Mean Optical Density (MOD) values. Scale Bar = 100μm. **(B)** Immunofluorescence detection of CCR1+ macrophage infiltration. Scale Bar = 20μm. Statistical analysis of the difference in CD68+CCR1+ cell numbers between high and low CCL16 expression groups using unpaired Student’s t-test. **: *P* < 0.01. Pearson correlation analysis of CCL16 expression and CCR1+ macrophage infiltration, r = 0.5743, *P* < 0.001.

In order to examine the involvement of the CCL16-CCR1 axis in clinical samples, a total of 42 liver cancer specimens were gathered. The specimens were subjected to immunohistochemical analysis to assess the expression of CCL16. The specimens were categorized into groups of high and low expression based on the median MOD value. **Table 3** provides a summary of the variations in clinical characteristics observed in both groups. The results of the chi-squared test indicated a notable correlation between the levels of CCL16 protein expression and advanced stage, as well as the presence of lymph node and distant metastasis. Additionally, immunohistochemical staining was performed to evaluate the presence of CCR1, CD68, and CD206 expression. Significant positive associations between CCL16 and the other three proteins were validated (**Figure 8A**). In addition, the proportions of CD68+CCR1+ macrophages were determined using immunofluorescence staining, revealing a robust positive association between CCL16 expression and infiltration of CD68+CCR1+ macrophages (**Figure 8B**). The clinical pathological analyses confirm that the CCL16-CCR1 axis has the capability to enhance the infiltration of M2 macrophages in the microenvironment of liver cancer, indicating that targeting the CCL16-CCR1 axis could be a promising therapeutic approach for liver cancer.

**Table 3:** Clinical characteristics of patients with high and low expression of CCL16.

Clinical Features	CCL16-Low (N=21)	CCL16-High (N=21)	*P*-value
Age			
≥60	7 (33.3%)	11 (52.4%)	0.35
<60	14 (66.7%)	10 (47.6%)	
Gender			
Male	12 (57.1%)	10 (47.6%)	0.757
Female	9 (42.9%)	11 (52.4%)	
Stage			
I+II	19 (90.5%)	6 (28.6%)	<0.001
III+IV	2 (9.5%)	15 (71.4%)	
T			
T2	2 (9.5%)	2 (9.5%)	1
T3+T4	19 (90.5%)	19 (90.5%)	
N			
N0	19 (90.5%)	6 (28.6%)	<0.001
N1+N2	2 (9.5%)	15 (71.4%)	
M			
M0	20 (95.2%)	14 (66.7%)	0.0494
M1	1 (4.8%)	7 (33.3%)	

## Discussion

Hepatocellular carcinoma (HCC), a prevalent and aggressive cancer, has a significant global impact and a high fatality rate (1). The interaction among cells facilitated by ligand-receptor complexes, which contributes to the diversity of the tumor microenvironment, has been acknowledged as a vital element impacting the prognosis of HCC patients and propelling tumor advancement (35). Song et al. provide a comprehensive perspective on analyzing the immune microenvironment in HCC and emphasize the presence of a unique subset of macrophages characterized by CCL18 and CREM expression, which is notably enriched in advanced HCC patients. This specific macrophage subset plays a significant role in driving tumor progression and holds promising potential for future immunotherapeutic strategies (36). Chen et al. examined the role of cancer-associated fibroblast (CAF)-induced M2-polarized macrophages in promoting the progression of HCC through the plasminogen activator inhibitor-1 pathway. Additionally, they assessed macrophage polarization and identified key paracrine factors involved in their interactions with both CAFs and cancer cells in the context of HCC (37). Li et al. made a significant finding demonstrating that targeting the CCL2/CCR2 axis therapeutically could effectively impede the recruitment of inflammatory monocytes, inhibit tumor-associated macrophage (TAM) infiltration, and reverse M2 polarization. Consequently, this intervention effectively counteracted the immune-suppressive conditions within the tumor microenvironment, subsequently activating anti-tumor CD8 T cell responses (38). Yang et al. found that upregulated CD36 in metastasis-associated macrophages (MAMs) promoted M2 polarization, facilitates liver cancer metastasis through interactions with tumor cells, and its loss in MAMs can attenuate liver metastasis in mice (39).

Chemokines derived from tumor cells play a crucial role in the complex tumor microenvironment, with dysregulated cytokine production in the tumor microenvironment influencing all stages of carcinogenesis and affecting cancer initiation, progression, and responses to therapy (40). Zha et al. discovered tumor cells utilized complement-derived C3 to inhibit antitumor immunity by regulating tumor-associated macrophages through the C3a-C3aR-PI3Kγ pathway (41). Park et al. employed quantitative proteomics to unveil that exosomes originating from tumor cells under hypoxic conditions exhibit a significant enrichment of immunomodulatory proteins and chemokines. This enrichment includes notable factors such as CSF-1, CCL2, FTH, FTL, and TGFβ, all of which contribute to the promotion of macrophage M2 polarization (42). In both mouse models and immunotherapy-treated patients, House et al. found that macrophages, as the main source of CXCL9, significantly upregulated the ligand of CXCR3, which, upon CXCL9 knockout, led to reduced CD8 T cell infiltration and compromised therapeutic efficacy of PD-1/CTLA-4 immune checkpoint blockade therapy (43). Korbecki et al. emphasized the significant role of human CC motif chemokine ligands and their corresponding receptors in mediating the chemotaxis of immune cells to the tumor microenvironment (44). However, the role of CCL16 in the hepatocellular carcinoma microenvironment remains to be further investigated. To uncover the underlying mechanisms, we conducted a series of experiments, including Transwell co-culture and migration assays, immunofluorescence, immunoprecipitation, flow cytometry, and immunohistochemistry. Specifically, in co-culture experiments, CCL16-overexpressing hepatocellular carcinoma cells promoted migration and M2 polarization of macrophages towards tumor cells. This effect was mediated by the interaction between CCL16 secreted by tumor cells and the CCR1 receptor on macrophages. In clinical tissue samples, we observed a significant positive correlation between CCL16 and CCR1, as well as CD206 and CD68 at the protein level. Furthermore, CCL16 high-expressing patient tissues showed a significant increase in CD68+CCR1+ cells, further validating the recruitment role of CCL16 in CCR1-positive macrophages. These experiments allowed us to elucidate the role of the CCL16-CCR1 axis in recruiting tumor-associated macrophages and promoting M2 polarization in HCC.

Using the CellChat algorithm, we conducted an analysis of single-cell transcriptomic data obtained from HCC tumor samples. Our primary objective was to investigate the interactions between different cell populations within the HCC microenvironment, with a specific focus on ligand-receptor interactions involving malignant HCC cells. In this context, we identified key genes, such as CCL16 and CCR1, that are instrumental in these interactions. Furthermore, we leveraged RNA-Seq data from comprehensive datasets, including TCGA-LIHC and LIRI-JP, and employed Cox regression analysis to uncover predictive genes. We also employed unsupervised clustering to categorize individuals into molecular subgroups. Additionally, we developed a risk model comprising four genes by analyzing differential gene expression among subtypes. This model can be utilized for predicting the prognosis of HCC patients. Moreover, through bioinformatics analysis of the immune microenvironment, we made the noteworthy observation that the subtype associated with the poorest prognosis exhibited a significant increase in the infiltration score of M2 tumor-associated macrophages.

Our study provides valuable insights into the molecular subtyping, prognostic modeling, and the immune microenvironment of HCC. We achieved this by characterizing interactions between ligands and receptors and uncovering the pro-tumorigenic role of the CCL16-CCR1 axis within the HCC microenvironment. The findings from this research could have implications for the development of novel treatment strategies targeting specific ligand-receptor interactions or the modulation of the immune microenvironment in HCC. It’s worth noting that our study did not involve animal trials due to the absence of a homologous gene in mice, which presents a significant limitation when investigating the role of CCL16 in the immune microenvironment. Consistent with this, a study published in the journal Cell demonstrated that CCL16, produced by hepatocytes, binds to CCR1 expressed by human Kupffer cells (KCs) but not murine KCs. In this context, KCs refer to hepatic macrophages. Therefore, human CCL16 cannot interact with murine macrophages through CCR1 (45). On the other hand, as the first-discovered C-C chemokine receptor, CCR1 is overexpressed in several types of cancers and is associated with increased immune-suppressive cell infiltration and tumor metastasis (46, 47). Through literature and patent searches, several CCR1 antagonists are currently in development, including AZD-4818, BI-638683, BL-5923, BX-471, C-6448, C-4462, CCX9588, CCX354, CCX721, CP-481715, MLN-3701, MLN-3897, PS-031291/PS-375179, and UCB-35625. However, as of now, none of them have entered clinical trials in the field of oncology (48). The selective CCR1 antagonist CCX721 has demonstrated efficacy in reducing tumor burden and osteolytic lesions in a murine model of multiple myeloma (MM) by blocking osteoclasts (49). Additionally, studies have reported that inhibiting CCR1 with the receptor antagonist BL5923 suppresses the recruitment of immature myeloid cells, leading to a reduction in metastatic colon cancer and a significant prolongation of survival in mice with colon cancer liver metastasis (50). The combination of the CCR1 antagonist CCX9588 with an anti-PD-L1 antibody has shown promise as a therapeutic approach, synergistically inhibiting primary tumor growth and lung metastasis in an *in situ* breast cancer mouse model (51). Recently, in a mouse model of ovarian cancer, the small-molecule CCR1 inhibitor UCB35625 demonstrated the ability to reduce cell migration to the greater omentum, a preferential metastatic site for such cancers (52). Overall, these findings suggest that targeting CCR1 is a viable therapeutic strategy capable of limiting metastasis and delaying disease progression.

## Data availability statement

The original contributions presented in the study are included in the article/**Supplementary Material**. Further inquiries can be directed to the corresponding author.

## Ethics statement

The studies involving humans were approved by the Ethics Committee at the First Hospital of China Medical University. The studies were conducted in accordance with the local legislation and institutional requirements. The participants provided their written informed consent to participate in this study.

## Author contributions

ZD: Conceptualization, Formal Analysis, Investigation, Writing – original draft. YW: Conceptualization, Formal Analysis, Resources, Writing – review & editing. NS: Conceptualization, Investigation, Validation, Writing – review & editing. CZ: Conceptualization, Resources, Supervision, Validation, Writing – review & editing.

## Supplementary material

Supplementary Figure 1 Analysis of immune microenvironment differences among the three molecular subtypes. **(A)** Comparison of differences in immune cell infiltration gene set scores obtained through ssGSEA method. **(B)** Comparison of differences in Stromal score and Immune Score obtained through Estimate algorithm. **(C)** Differential expression of immune checkpoint genes among the three subtypes. **(D)** Comparison of differences in immune feature scores calculated by TIDE tool. Wilcoxon test. ns, not significant; *: *P*<0.05; **: *P*<0.01; ***: *P*<0.001; ****: *P*<0.0001.

Supplementary Figure 2 Identification of differentially expressed genes among three subtypes. **(A)** Volcano plots showing differential expression analysis using the Limma R package to identify genes that are differentially expressed between each group and the other two groups. **(B)** Functional enrichment analysis results of the commonly differentially expressed genes.

Supplementary Figure 3 Differential analysis of the immune microenvironment between high and low-risk groups. **(A)** There is a significant negative correlation between the risk score and immune score (R = -0.23, P = 6.4e-6). **(B)** Differences in Tumor-Reactive T Cell Signature (TRS score), cytolytic activity score (CYT score), and Th1/IFNγ gene signature score between high and low-risk groups. **(C)** Differential analysis of the scores of 28 immune cell gene sets between the two groups. Statistical analysis was performed using Wilcoxon test. ns, not significant; *: *P* < 0.05; **: *P* < 0.01; ***: *P* < 0.001; ****: *P* < 0.0001.

Supplementary file 1 The receptor-ligand pairs and pathway enrichment results for the interactions between different cell types, as identified by CellChat.

Supplementary file 2 The differential expression genes and functional enrichment results among the three molecular subtypes.

Supplementary file 3 The results of univariate Cox analysis in the TCGA-LIHC cohort and risk scores of patients in both cohorts.
