# Supplementary figures and images for: Characterizing ligand-receptor interactions and unveiling the pro-tumorigenic role of CCL16-CCR1 axis in the microenvironment of hepatocellular carcinoma
Source: Front Immunol. 2024 Jan 11;14:1299953. doi: 10.3389/fimmu.2023.1299953 (PMC10808667; doi:10.3389/fimmu.2023.1299953)

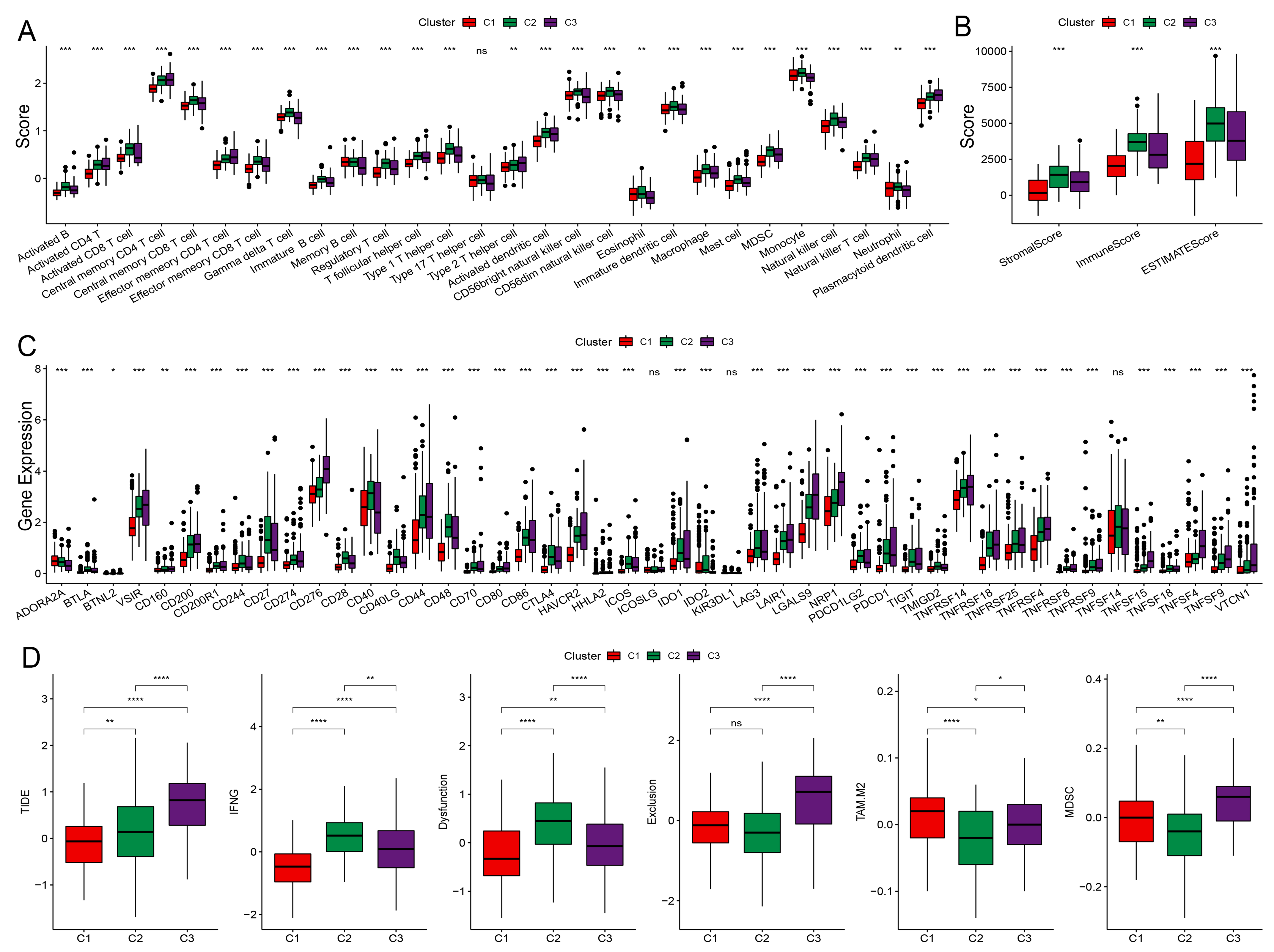

Supplement: Supplementary Figure 1 — Analysis of immune microenvironment differences among the three molecular subtypes. (A) Comparison of differences in immune cell infiltration gene set scores obtained through ssGSEA method. (B) Comparison of differences in Stromal score and Immune Score obtained through Estimate algorithm. (C) Differential expression of immune checkpoint genes among the three subtypes. (D) Comparison of differences in immune feature scores calculated by TIDE tool. Wilcoxon test. ns, not significant; *: P<0.05; **: P<0.01; ***: P<0.001; ****: P<0.0001. [file Image_1.tif]

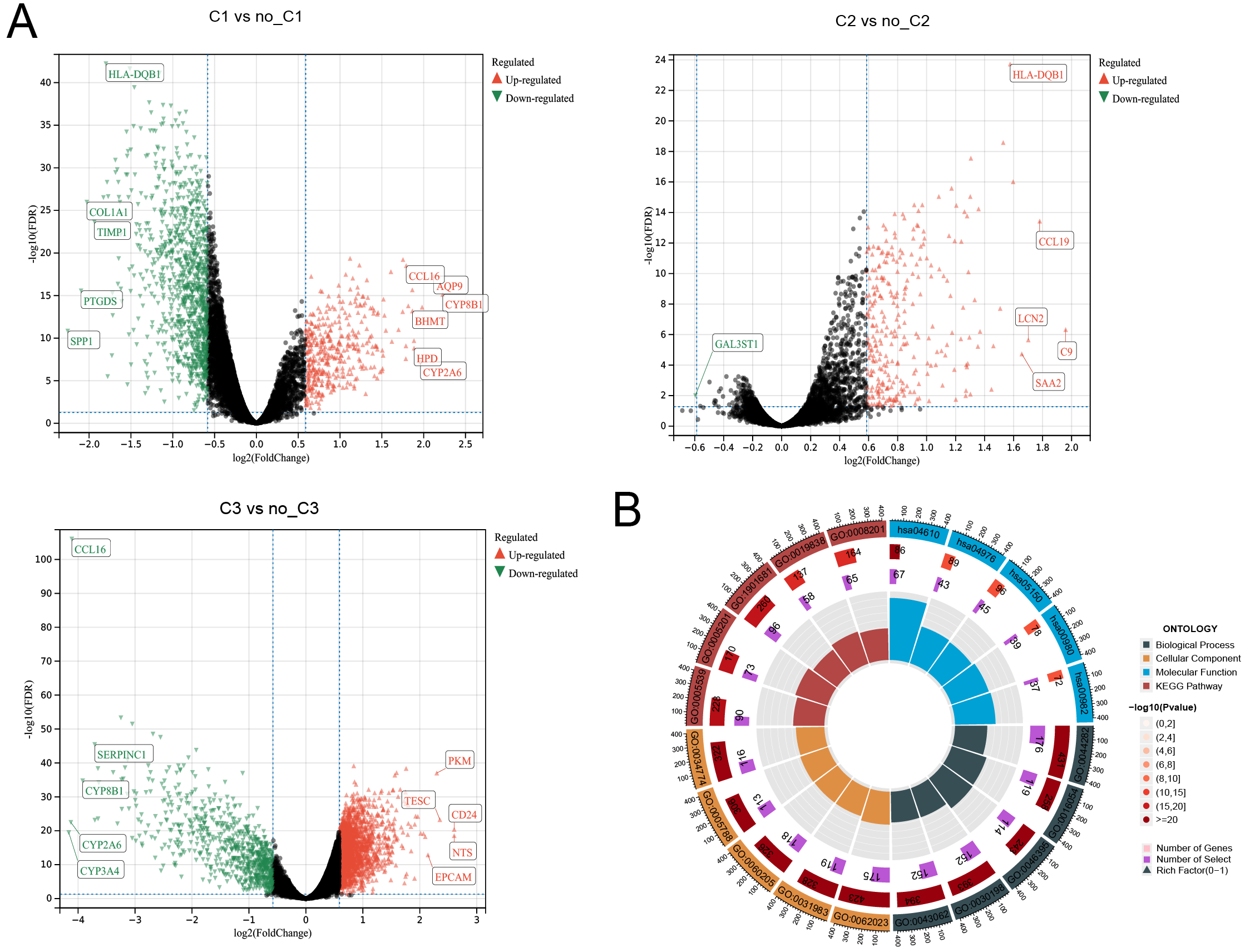

Supplement: Supplementary Figure 2 — Identification of differentially expressed genes among three subtypes. (A) Volcano plots showing differential expression analysis using the Limma R package to identify genes that are differentially expressed between each group and the other two groups. (B) Functional enrichment analysis results of the commonly differentially expressed genes. [file Image_2.tif]

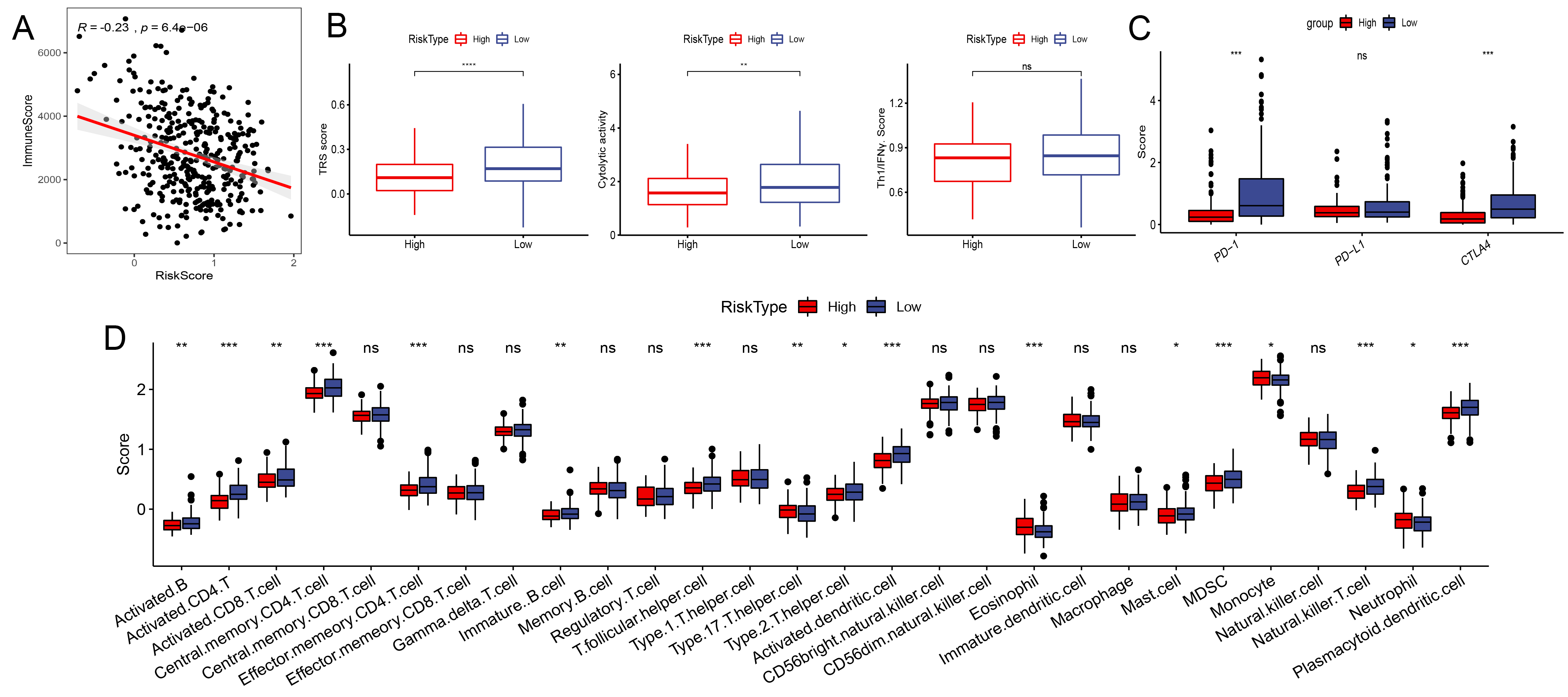

Supplement: Supplementary Figure 3 — Differential analysis of the immune microenvironment between high and low-risk groups. (A) There is a significant negative correlation between the risk score and immune score (R = -0.23, P = 6.4e-6). (B) Differences in Tumor-Reactive T Cell Signature (TRS score), cytolytic activity score (CYT score), and Th1/IFNγ gene signature score between high and low-risk groups. (C) Differential analysis of the scores of 28 immune cell gene sets between the two groups. Statistical analysis was performed using Wilcoxon test. ns, not significant; *: P < 0.05; **: P < 0.01; ***: P < 0.001; ****: P < 0.0001. [file Image_3.tif]
